# Dynamics of Experience in a Learning Protocol: A Case Study in Climbing

**DOI:** 10.3389/fpsyg.2020.00249

**Published:** 2020-02-20

**Authors:** Nadège Rochat, Guillaume Hacques, Caroline Ganière, Ludovic Seifert, Denis Hauw, Pierpaolo Iodice, David Adé

**Affiliations:** ^1^Center for the Study and the Transformation of Physical Activities, Faculty of Sport Sciences, University of Rouen Normandie, Rouen, France; ^2^Institute of Sport Sciences of the University of Lausanne, Lausanne, Switzerland

**Keywords:** learning, enaction, phenomenology, climbing, embodiment

## Abstract

Many of the studies on motor learning have investigated the dynamics of learning behaviors and shown that the learning process is non-linear, self-organized, and situated. Aligned with this research trend, studies within the enactive paradigm focus on learners’ lived experience to understand how it shapes their intentions, actions, and perceptions. Thus, a joint analysis of experiential and behavioral assessments might help to explain the dynamics of learning (e.g., the transition between stable states). The aim of this case study was to analyze the dynamics of a beginner climber’s lived experience as his performance progressed (i.e., climbing fluency) during a learning protocol. The protocol comprised 10 climbing sessions over 5 weeks. During the sessions, the climber had to climb a “control route” (CR) (i.e., a route that never changed) and “variants” (i.e., novel routes, in which the spatial layout of the holds was modified). Phenomenological data were collected with self-confrontation interviews after each session. From the verbalizations, a thematic analysis of the climber’s intentions, actions, and perceptions was performed to detect the general dimensions of his experience. The behavioral data (the climber’s performance) were assessed using four indicators of climbing fluency: climbing time (CT), immobility ratio (IR), geometric index of entropy (GIE) of the hip trajectory, and the jerk. Our results highlighted the dynamics of the climber’s lived experience and performances in the unchanged and novel environments. The dynamics on the CR were characterized by four crucial episodes and the dynamics on the variants, by four ways of experiencing novelty. Our results are discussed around three points: (i) the climber’s definition of his enacted fluency in terms of intentions, actions, and perceptions; (ii) how the definition was identified through a dynamic phenomenological synthesis; and (iii) three effects that characterize the dynamics: challenge, metaphor, and a refinement in perceptions.

## Introduction

### Grasping the Complexity of Learning Through Behavioral Modifications

The embodied approaches to motor learning conceive of learners as complex neurobiological systems (Chow et al., 2009) showing intrinsic self-organizing dynamics that constitute a repertoire of stable and spontaneous behaviors (Kostrubiec et al., 2012). This repertoire enables learners to adapt effectively to environmental and task constraints (Newell, 1986). From this perspective, learning is the reorganization of stable behaviors, with intrinsic dynamics perpetually being destabilized and reorganized as a function of the constraints (Zanone and Kelso, 1992). Learning in this sense is characterized by the non-linear dynamics of transitions from one stable coordination pattern to another (Schöner et al., 1992). During these transitions, new behaviors may temporarily alternate with previous behaviors in a so-called “intermittent regime” and are accompanied by an interplay of regressions and progressions in “measurable” performance (Nourrit et al., 2003; Teulier and Delignières, 2007). These periods of intermittent regime are nevertheless functional as they indicate the learner’s exploration and exploitation of new coordination solutions to achieve the task (Chow et al., 2011; Komar et al., 2019). This perspective led to the development in non-linear pedagogy (Chow, 2013) using a constraints-led approach (Davids et al., 2008), where teachers and/or practitioners manipulate a set of constraints (i.e., environment and task) in order to prompt improvements in the quality of learners’ explorations and thereby provoke the development of adaptable behaviors (Komar et al., 2019). In addition, the evidence is growing that learning is characterized by individual pathways in the interactions with constraints (Pacheco et al., 2017; Pacheco and Newell, 2018). These insights from dynamical systems theory and non-linear pedagogy suggest questions about whether learners live meaningful perturbations during the intermittent regimes or instead perceive a step-by-step or linear progression. In addition, it can be assumed that each individual has his/her own way of experiencing constraints and appropriating the design of the task. Individualized pathways can therefore be investigated at the level of the learner’s lived experience, as proposed by the enactive paradigm, which conceives individuals as embodied agents who meaningfully interact with the environment (Di Paolo, 2009).

### Learning as a Lived Experience: Shedding Light on Meaningful Transformations

The enactive paradigm (Varela et al., 1991; Stewart et al., 2010) conceives human cognition as consisting of the construction of significations that emerge from the continuous interaction between an actor and his/her environment (i.e., actor–environment coupling). These significations refer to the actor’s point of view about the environment, with which he/she interacts in function of his/her past experiences and his/her involvement in the situation. While the enactive paradigm shares the ideas of self-organization, non-linearity, and constraints traditionally defended by dynamical systems theory, it proposes a conception of cognition that is rooted in action, meaning that an actor brings forth (i.e., enacts) a meaningful situation from his/her coupling with the environment. Specifically, it conceives the actor–environment coupling as asymmetrical because the relationship with the environment is partly regulated by the actor’s lived experience. The core ideas about cognition within the enactive framework suggest that cognition can be investigated as: (i) fundamentally embedded in an environment in which constraints need to be regulated by the cognitive system; (ii) autonomous, as it emerges from ongoing structural couplings with the environment that define its own organization; (iii) embodied, which means that the bodily structures are key components of perceptions and actions; and (iv) regulated by a sense-making process, which means that the agent generates meaning and “casts a web of significance on [the] world” (Di Paolo et al., 2011, p. 39). To grasp how the actor builds meaning, we focus on pre-reflective consciousness, defined by Theureau (2006) as the level of consciousness at which an actor can show, mime, simulate, tell about, and comment on his/her activity in favorable conditions. In sports psychology, the potential of analyzing athletes’ activity via their pre-reflective consciousness has resulted in a growing number of studies (Sève et al., 2005; Hauw and Durand, 2007; Mohamed et al., 2015; Rochat et al., 2017) that provide practical recommendations for preparing, training, and managing competitions. We may thus expect that this approach applied to the learning process will yield practical recommendations for designing learning environments. Indeed, the analysis of lived experience has been shown to be helpful for designing computer-assisted learning environments (Leblanc et al., 2001), professional training and lifelong education programs (Durand, 2015), and elite sports preparation (Durand et al., 2005; Hauw, 2018).

### Lived Experience and Behavior as Complementary Domains of Evidence to Understand the Dynamics of Learning

Varela and Shear (1999) stated that lived experience is irreducible, which means that the pre-reflective phenomena that are brought forth by the actor “cannot be reduced or derived from the third-person perspective” (p. 4). Lived experience (i.e., the first-person) is thus in itself a domain of evidence just as much as the behavioral (i.e., third-person) domain. These authors therefore proposed that the circulation between the first- and third-persons would provide reciprocal contributions. From this perspective, accessing learners’ subjectivity at the phenomenological level would help to reconstruct their meaningful activity (i.e., their intentions, interpretations of the situation, focalizations, emotions, significations given to the unfolding action and the situation), and this would be accomplished in a mutually enriching relationship with the third-person indicators. Such phenomenological approaches have indeed been used to highlight that the dynamics of performers’ lived experience and behavior may be either convergent or divergent. For example, Sève et al. (2013) studied the lived experiences of rowers and identified their difficulties in synchronizing with their partners. These authors further found that the difficulties converged with kinematic measures indicating differences in stroke amplitudes and angular velocities between the rowers. A swimming study on the use of an underwater technical device (i.e., the MAD system) highlighted divergences between what was lived by the swimmers and the biomechanical measures of their movement (i.e., the force applied by the hands on the pads) (Gal-Petitfaux et al., 2013). In both these examples, the phenomenological data provided access to the way the actors created meaning about their activity and, when applied to learning, such data might well reveal the exploration strategies undertaken by learners. This in turn would help assess the effectiveness of exploration strategies and their impact on the intermittent regimes.

In the present study, we jointly investigated a learner’s lived experience and performances to (i) enrich the analysis of learners’ experience with third-person data in order to objectivize the transformations linked to learning, and (ii) examine the possible differences/divergences between what is objectivized and what the actor lives. To attain this goal, we used a phenomenological approach that attempts to address the complexity of the non-linear dynamics of learning by giving importance to what the learners live without, however, excluding the third-person data that provide access to the intermittent regimes. Therefore, the present study, which is based on the principles of self-organization and non-linear dynamics, seeks to understand how these principles manifest in a learning situation in climbing, mainly from a phenomenological and enactive point of view but not exclusively. We propose a case study of a beginner climber following a learning protocol characterized by variable practice (i.e., climbing on unchanged and novel climbing routes) in order to characterize how he deals with the novelty of the environment. During the protocol, we followed his lived experience at the pre-reflexive level in relation to his performances (i.e., his climbing fluency). By doing so, we sought to understand how he embodied climbing fluency in terms of intentions, actions, perceptions, and performances. In sum, we sought to model the phenomenology of learning dynamics as a function of the novelty of the encountered situations and propose an integrative approach that articulates lived experience and recorded behaviors during the performance of a climbing task: climbing the whole route as fluently as possible with variable practice (i.e., novelty during learning when the climbing route is changed). We assumed that the results would have a twofold impact: first, for the development of integrative research methods to investigate learning, where learners’ points of view are taken into account, and second, for designing appropriate learning situations to prompt beginners to effectively explore their environment.

## Materials and Methods

### Research Design

The present research is a case study that mobilizes a methodology that is able to pick up the modifications in a learner’s lived experience in relation to the progress in his performances throughout a learning protocol. By considering the phenomenon under study as inseparable from its context, we expected to provide a detailed analysis of (i) what is typical, recurrent, and crucial in the learner’s experience and (ii) the transformations in the learner’s own world (i.e., the meaningful situation enacted by him/her) from a dynamical and situated perspective. Hence, our intention was not to generalize the results stemming from this analysis, but rather to propose a methodology integrating two domains of evidence (i.e., for future research, this type of analysis might be feasible for larger samples of participants and for other learning protocols).

### Participant

The participant was a 20-year male volunteer (height: 174.5 cm, weight: 66 kg, arm span: 178 cm, right-handed). He was an undergraduate student at the sports faculty of Rouen Normandy University but had no prior experience in climbing. The protocol was explained to the participant and he gave his written informed consent before starting the experiment.

### Protocol

The protocol was composed of 10 climbing sessions (i.e., two sessions per week) on an artificial wall in the gym facilities of Rouen Normandy University. This artificial wall had three routes dedicated to the protocol. One route, which never changed, was called the “control route” (CR) and was 525 cm high with 20 holds (i.e., 13 handholds and 7 footholds). The CR was the unchanged environment. The first three climbs of every session were performed on this route. The other two routes were on a wall 480 cm high and each also had 20 holds. These two routes were called “variants” (V) as they changed every two sessions. A total of nine variants were applied during the protocol. Six trials per variant were performed during two successive sessions (Table 1). These variants were the novel environments. A total of 30 trials were performed on the CR and 54 on the variants. Importantly, when designing the variants, we changed the spatial layout of the handholds but did not manipulate the difficulty of the routes. Also, the shape of the holds was always the same. When the other two routes were not used, they were covered with a tarpaulin so that only the route to be climbed was visible. Before each climbing session, the participant had a 10-min warm-up on easy boulder routes in the bouldering area of the climbing gym. Before each climb, the climber had a 30-s preview that he could use completely or not. Prior to each climb, the following instructions were given: “Use all the handholds in a bottom-up order, do not to use holds with both hands or both feet at the same time. In addition, you have to find a way to climb the route as fluently as possible: that is, avoiding pauses and saccades.” At the beginning of each session before the warm-up, the climber was given feedback about his climbing fluency (see the section “Behavioral Data Processing” for more detail). Each session lasted 1 h on average, including the warm-up, equipping the participant, giving him the instructions before each trial with previews and climbs, and launching the cameras and the inertial measurement unit (IMU) recordings for each trial (see the section “Behavioral Data Collection” for more detail). The participant also performed a transfer route in sessions 1 and 10, but these results are not presented for the present study, as we focus here on the learning dynamics and not on what was transferred between pre- and post-learning.

**TABLE 1:** Organization of the learning protocol.

**Week 1**		**Week 2**		**Week 3**		**Week 4**		**Week 5**	
**Session 1**	**Session 2**	**Session 3**	**Session 4**	**Session 5**	**Session 6**	**Session 7**	**Session 8**	**Session 9**	**Session 10**
10-min warm-up	10-min warm-up	10-min warm-up	10-min warm-up	10-min warm-up	10-min warm-up	10-min warm-up	10-min warm-up	10-min warm-up	10-min warm-up
1xTR	3xCR	3xCR	3xCR	3xCR	3xCR	3xCR	3xCR	3xCR	3xCR
3xCR	3xV1	3xV2	3xV3	3xV4	3xV5	3xV6	3xV7	3xV8	3xV9
3xV1	3xV2	3xV3	3xV4	3xV5	3xV6	3xV7	3xV8	3xV9	1xTR
Interview	Interview	Interview	Interview	Interview	Interview	Interview	Interview	Interview	Interview

*This table presents the organization of each session (1–10) of the learning protocol. Each session contained a 10-min warm-up and was followed by an interview. Each square shows the number of trials performed on the different routes: control route (CR); variant 1 (V1); variant 2 (V2), etc. In the first and last trials, the participant performed a transfer route (TR).*

### Data Collection

#### Phenomenological Data Collection

Phenomenological data were collected immediately after each session with self-confrontation interviews based on the video recordings of each preview and climb (Hauw and Durand, 2007; Adé et al., 2017). These video recordings were used as past-activity traces to help the climber re-enact his past experience as it emerged during the climbs. This means that he was invited to chronologically relive his meaningful experience throughout the climbing trials. On the basis of these traces, the interviews consisted of asking him to comment his activity during the previews and climbs, avoiding retrospective judgments and generalizations. Especially, the interview prompts aimed to document (i) the climber’s intentions (what are you trying to do?), (ii) his actions (what are you doing?), and (iii) his perceptions (what is drawing your attention? What are you feeling?). The interviews were conducted by two trained researchers who were experienced in conducting self-confrontation interviews with athletes from different sports. In total, 10 1.15-h interviews were conducted for the present study.

#### Behavioral Data Collection

Behavioral data were collected for each climb to compute the climber’s fluency scores. The participant wore a light and an IMU (HIKOB FOX^®^, Villeurbanne, France) on the back of his harness. The sensor recorded the signal from an accelerometer, a gyroscope, and a magnetometer at 100 Hz. Ascents were filmed at 29.97 fps on 1920 × 1080 pixel frames with GoPro Hero 5^®^ (GoPro Inc., San Mateo, CA, United States) cameras covering each entire wall (i.e., one camera per wall). The holds of the route were instrumented with the Luxov^®^ Touch system^1^ (Arnas, France) that uses a capacitive sensing technology to detect and record the time of contact on each hold. For the present study, these data (i.e., the signal of the touch) was used only to compute the climbing time (CT) (from the climber’s first movement – either with a hand or foot – from the starting position until he touched the last handhold).

### Data Processing

The data were processed in two successive steps: the first step consisted of separate processing of the phenomenological data and the behavioral data, and the second step consisted of portraying the learning dynamics from the combination of these two types of data.

#### Phenomenological Data Processing

**FIGURE 1:**
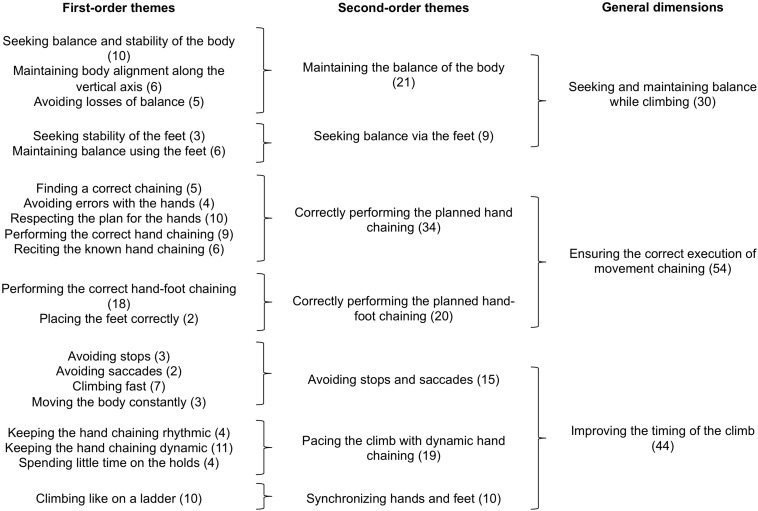
Thematic analysis of the climber’s intentions. The first column presents the first-order themes. The second column presents the second-order themes (i.e., merging of the first-order themes). The third column presents the general dimensions (i.e., merging of the second-order themes). The numbers in parentheses associated with each theme refer to the number of occurrences identified from the verbalizations.

**FIGURE 2:**
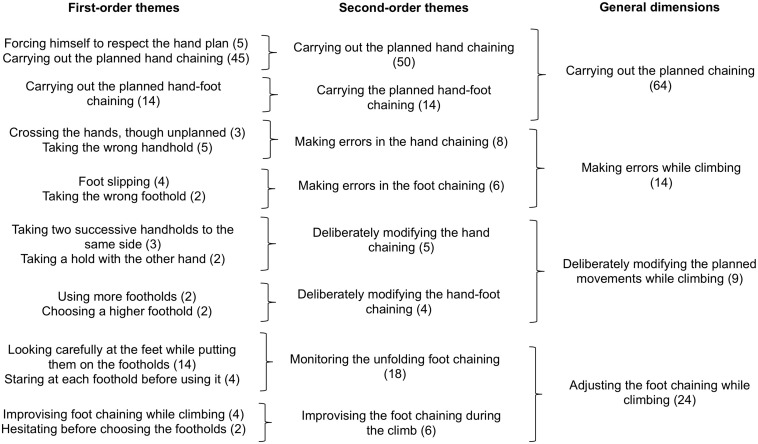
Thematic analysis of the climber’s actions. The first column presents the first-order themes. The second column presents the second-order themes (i.e., merging of the first-order themes). The third column presents the general dimensions (i.e., merging of the second-order themes). The numbers in parentheses associated with each theme refer to the number of occurrences identified from the verbalizations.

**FIGURE 3:**
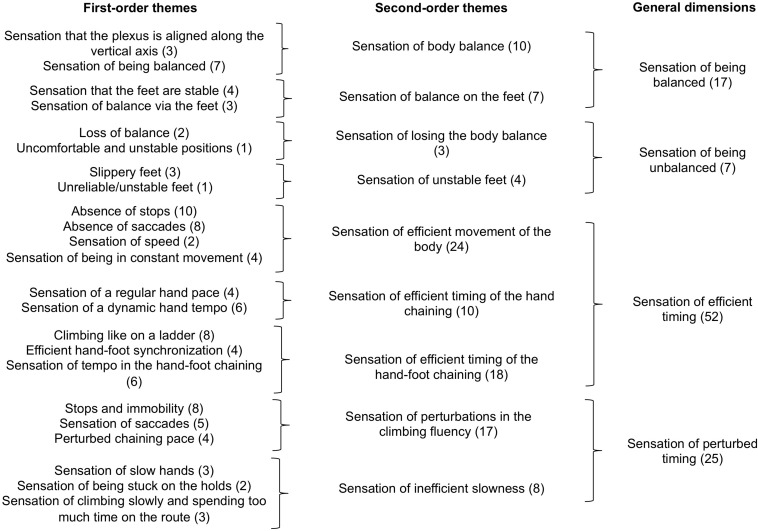
Thematic analysis of the climber’s perceptions. The first column presents the first-order themes. The second column presents the second-order themes (i.e., merging of the first-order themes). The third column presents the general dimensions (i.e., merging of the second-order themes). The numbers in the parentheses associated with each theme refer to the number of occurrences identified from the verbalizations.

**FIGURE 4:**
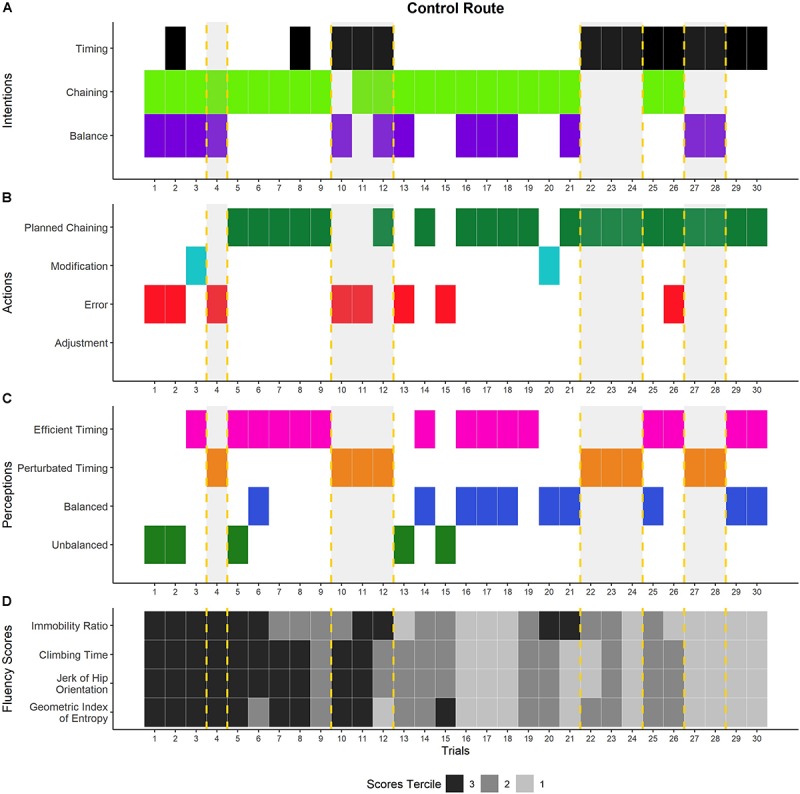
Dynamics of the climber’s experience and performances on the control route. Panels **A–C** show the temporal organization of the general dimensions of the different components of experience detected during the interviews. Panel **A** shows the general dimensions of the climber’s intentions: Improving the timing of the climb (black), ensuring the correct execution of movement chaining (light green), and seeking and maintaining balance while climbing (purple). The climber’s actions are shown in panel **B:** carrying out the planned chaining (dark green), deliberately modifying the planned movements while climbing (light blue), making errors while climbing (red), and adjusting the foot chaining while climbing (gold). Panel **C** shows the general dimensions of the climber’s perceptions: sensation of efficient timing (pink), sensation of perturbed timing (orange), sensation of being balanced (dark blue), and sensation of being unbalanced (green). Panel **D** shows the score terciles for each fluency indicator. The climbing performance is expressed in the terciles: tercile 3 (in black) refers to his poorest performances, tercile 2 (in dark gray) refers to his intermediate performances, and tercile 1 (in light gray) refers to his best performances. The vertical dashed lines delimit the crucial episodes in the climber’s experience.

**FIGURE 5:**
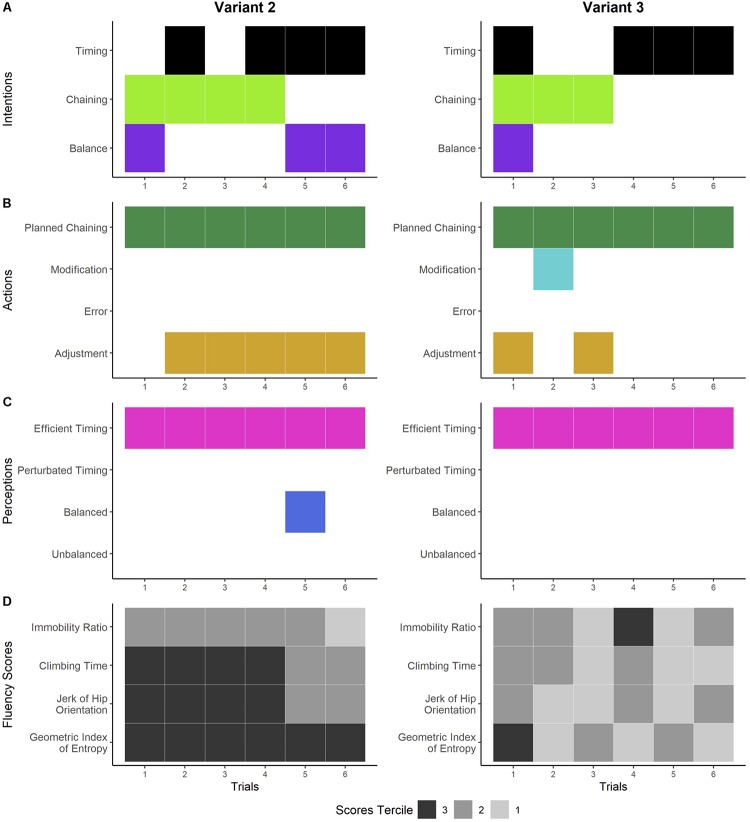
Dynamics of the climber’s experience and performances on variants 2 and 3. Panels **A–C** show the temporal organization of the general dimensions of the different components of experience detected during the interviews. Panel **A** shows the general dimensions of the climber’s intentions: improving the timing of the climb (black), ensuring the correct execution of movement chaining (light green), and seeking and maintaining balance while climbing (purple). The climber’s actions are shown in panel **B:** Carrying out the planned chaining (dark green), deliberately modifying the planned movements while climbing (light blue), making errors while climbing (red), and adjusting the foot chaining while climbing (gold). Panel **C** shows the general dimensions of the climber’s perceptions: sensation of efficient timing (pink), sensation of perturbed timing (orange), sensation of being balanced (dark blue), and sensation of being unbalanced (green). Panel **D** shows the score terciles for each fluency indicator. The climbing performance is expressed in the terciles: tercile 3 (in black) refers to his poorest performances, tercile 2 (in dark gray) refers to his intermediate performances, and tercile 1 (in light gray) refers to his best performances.

**FIGURE 6:**
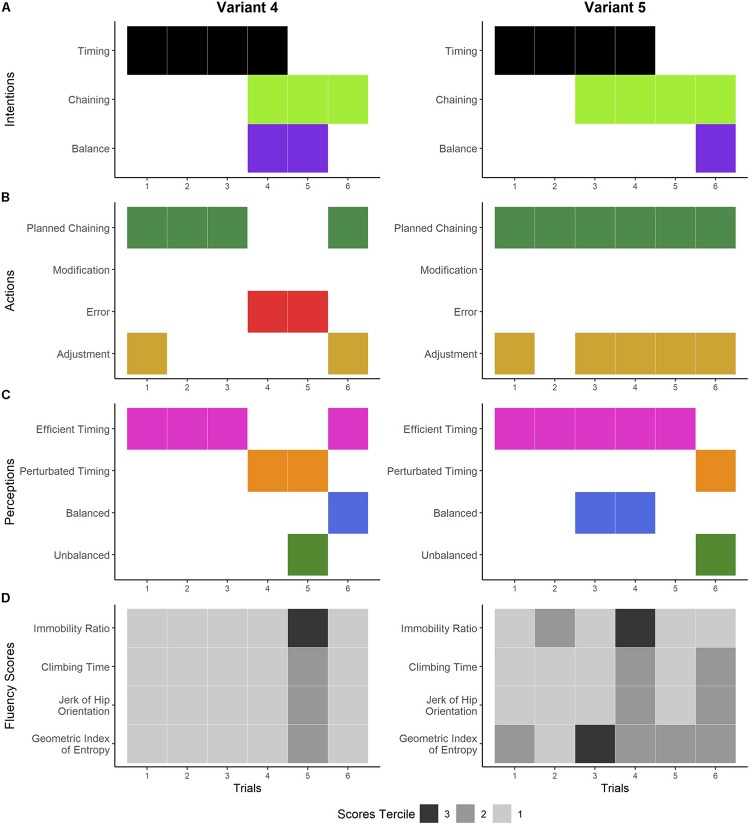
Dynamics of the climber’s experience and performances on variants 4 and 5. Panels **A–C** show the temporal organization of the general dimensions of the different components of experience detected during the interviews. Panel **A** shows the general dimensions of the climber’s intentions: improving the timing of the climb (black), ensuring the correct execution of movement chaining (light green), and seeking and maintaining balance while climbing (purple). The climber’s actions are shown in panel **B:** carrying out the planned chaining (dark green), deliberately modifying the planned movements while climbing (light blue), making errors while climbing (red), and adjusting the foot chaining while climbing (gold). Panel **C** shows the general dimensions of the climber’s perceptions: sensation of efficient timing (pink), sensation of perturbed timing (orange), sensation of being balanced (dark blue), and sensation of being unbalanced (green). Panel **D** shows the score terciles for each fluency indicator. The climbing performance is expressed in the terciles: tercile 3 (in black) refers to his poorest performances, tercile 2 (in dark gray) refers to his intermediate performances, and tercile 1 (in light gray) refers to his best performances.

**FIGURE 7:**
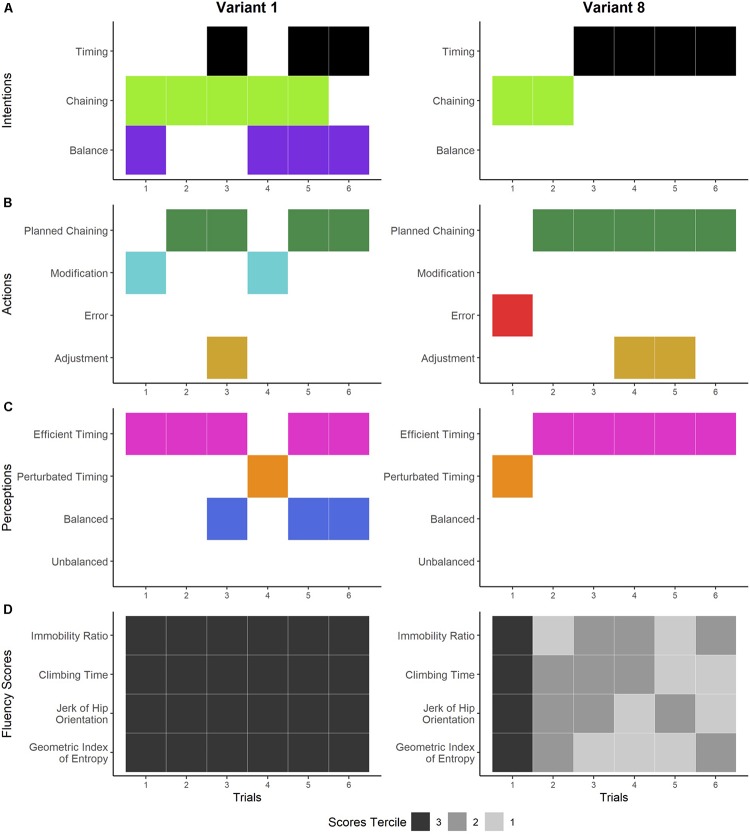
Dynamics of the climber’s experience and performances on variants 1 and 8. Panels **A–C** show the temporal organization of the general dimensions of the different components of experience detected during the interviews. Panel **A** shows the general dimensions of the climber’s intentions: seeking and maintaining balance while climbing (purple), ensuring the correct execution of movement chaining (light green), and improving the timing of the climb (black). The climber’s actions are reported in panel **B:** adjusting the foot chaining while climbing (gold), making errors while climbing (red), deliberately modifying the planned movements while climbing (light blue), and carrying out the planned chaining (dark green). Panel **C** shows the general dimensions of the climber’s perceptions: sensation of being balanced (dark blue), sensations of being unbalanced (green), sensations of efficient timing (pink), and sensations of perturbed timing (orange). Panel **D** shows the score terciles for each fluency indicator. The climbing performance is expressed in the terciles: tercile 3 (in black) refers to his poorest performances, tercile 2 (in dark gray) refers to his intermediate performances, and tercile 1 (in light gray) refers to his best performances.

**FIGURE 8:**
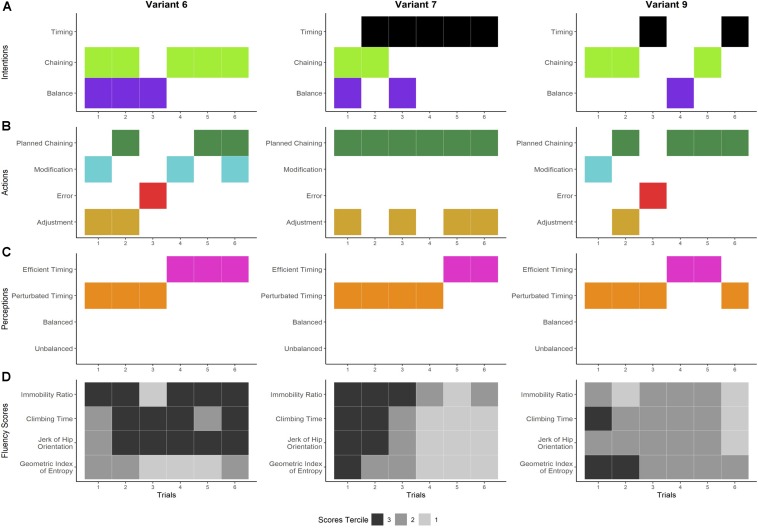
Dynamics of the climber’s experience and performances on variants 6, 7, and 9. Panels **A–C** show the temporal organization of the general dimensions of the components of experience detected during the interviews. Panel **A** shows the general dimensions of the climber’s intentions: seeking and maintaining balance while climbing (purple), ensuring the correct execution of movement chaining (light green), and improving the timing of the climb (black). The climber’s actions are shown in panel **B:** Adjusting the foot chaining while climbing (gold), making errors while climbing (red), deliberately modifying the planned movements while climbing (light blue), and carrying out the planned chaining (dark green). Panel **C** shows the general dimensions of the climber’s perceptions: sensation of being balanced (dark blue), sensation of being unbalanced (green), sensation of efficient timing (pink), and sensation of perturbed timing (orange). Panel **D** shows the score terciles for each fluency indicator. The climbing performance is expressed in the terciles: tercile 3 (in black) refers to his poorest performances, tercile 2 (in dark gray) refers to his intermediate performances, and tercile 1 (in light gray) refers to his best performances.

The phenomenological data were processed in three steps: (i) semiotic labeling of the climber’s course of experience, (ii) thematic analysis of the course of experience, and (iii) analysis of the phenomenological dynamics. Each step was co-jointly performed by the first, third, and last authors, who were all trained in performing this type of phenomenological data processing. In case of disagreement, the researchers re-watched the video recordings of the self-confrontation interviews and the climbs and debated until a consensus was found. First, we restored the climber’s course of experience for each of his 84 climbs. This consisted of a semiotic labeling of his intentions, perceptions, and actions from his verbalizations in the self-confrontation interviews and the climbing activity recorded on the videos [as already done in Rochat et al. (2018), for trail running and Seifert et al. (2017), for rowing, for example]. Second, similar to R’Kiouak et al. (2016), we conducted a thematic analysis to inductively find similarities in the climber’s intentions, actions, and perceptions (Braun and Clarke, 2006; Vaismoradi et al., 2013) in order to characterize the general dimensions that made up his course of experience and his definition of his enacted fluency. The raw data of the intentions, actions, and perceptions were examined in detail and the detection of similarities among them helped identify the first-order themes, which were merged into second-order themes, and then general dimensions. The identified themes enabled us to inductively characterize the general dimensions, which indicated the modalities by which fluency was meaningfully enacted in terms of intentions, actions, and perceptions (Figures 1–3). For this stage of the processing, we applied thematic analysis to each category of the course of experience (i.e., the intentions, actions, and perceptions) separately to arrive at an intelligible synthesis of the elements that were meaningful for the climber. Third, to characterize the phenomenological dynamics on the CR and on each of the nine variants, we (i) reconstructed the climber’s courses of experience with the combination of the general dimensions of the intentions, actions, and perceptions for each trial, and (ii) tracked the cumulative sum of each general dimension throughout the trials in order to obtain their dynamics of emergence. The processing was done on RStudio 1.1.383 that used R version 3.5.1 (Figures 4–8).

#### Behavioral Data Processing

To assess the climbing performance of each trial, we relied on four indicators that included CT (i.e., from the moment when the climber touched another hold from that of the initial position to the moment when the last handhold was touched) and three fluency indicators: (i) the immobility ratio (IR), which is a temporal indicator that refers to the percentage of CT spent immobile (Seifert et al., 2015; Orth et al., 2018), (ii) the geometric index of entropy (GIE) of the hip trajectory, which is a spatial indicator that refers to “the degree of coherence of the action–perception coupling” (Cordier et al., 1994, p. 748), and (iii) the jerk of hip orientation, which is a spatiotemporal indicator (Seifert et al., 2014). By combining these indicators, we sought to determine whether climbing fluency was impacted by spatial factors (i.e., searching the route), temporal factors (i.e., searching the best way to grasp a hold), or spatiotemporal factors (i.e., transiting between holds). The percentage of CT spent immobile was based on a hip velocity threshold (set at 20 cm/s) (Orth et al., 2018, p. 8). The GIE refers to the length of the hip trajectory divided by the length of its convex hull to which a logarithmic transformation is applied. This index reflects the complexity of the hip trajectory. To assess the GIE, the harness light was video-tracked on Kinovea© software (version 0.8.25, Boston, MA, United States) to obtain the climber’s coordinate of hip trajectory projection on the 2D wall. The camera lens distortion was compensated by importing the intrinsic parameters of the camera, and the video perspective was corrected using a manually set grid-based calibration on this software. The jerk of hip orientation corresponds to the third derivative of the hip orientation and informs about the spatiotemporal fluency (Seifert et al., 2014). All processing was performed with MATLAB R2014a^®^ software (version 8.3.0.532, The MathWorks Inc., Natick, MA, United States). The scores for spatial immobility, GIE, and jerk for each climb were given as feedback to the participant in the subsequent climbing session (e.g., the scores in session 1 were given at the beginning on session 2 and so on). From the score values, three terciles were computed to express the climber’s performance. The values included in the first tercile corresponded to his best scores, the values in the second tercile corresponded his intermediate scores, and the values in the third tercile corresponded to his poorer scores. It must be noted that for the four indicators of fluency, the lower the scores were, the better the performance was, and inversely. In order to obtain a more accurate delimitation of the terciles, the computations were conducted on the CR and variants separately. Therefore, each tercile contained the ranges of values displayed in Table 2.

**TABLE 2:** The interval values identifying the terciles for the geometric index of entropy (GIE), jerk, immobility ratio (IR, in percentage), and climbing time (CT, in seconds).

**Tercile**	**Trials (*N*)**	**GIE**	**Jerk**	**IR (%)**	**CT (s)**
**Control route**					
1	10	0.55–0.72	10.28–11.48	6.99–16.64	8.86–11.17
2	10	0.72–0.85	11.48–12.34	16.64–23.72	11.17–13.56
3	10	0.85–1.40	12.34–16.00	23.72–42.42	13.56–38.05
**Variants**					
1	18	0.58–0.67	10.53–11.37	7.17–15.09	8.79–10.72
2	18	0.67–0.78	11.37–12.19	15.09–20.10	10.72–13.11
3	18	0.78–1.25	12.19–14.98	20.10–42.63	13.11–29.18

*All interval values are presented for the control route and the nine variants (all together). For tercile 1, the minimal values correspond to his absolute best scores, while the maximal values of tercile 3 correspond to his absolute worst scores.*

#### Characterization of the Dynamics of the Phenomenological and Behavioral Data: A First-Person Entry

In this step of the data processing, the dynamics of emergence of the general dimensions identified in the phenomenological data were synchronized with the progression in the fluency scores on CR and each variant separately. This step had a twofold aim: (i) to characterize the salient episodes that made up the learning dynamics in the unchanged and novel environments, and (ii) to determine the congruence/divergence between the climber’s experience and his actual performance scores. We therefore focused on when the climber perceived a meaningful perturbation in his climbing fluency, and we qualitatively characterized each episode on the basis of his verbalizations during the interviews. We then took into account the scores in these episodes to investigate whether they were congruent or divergent with his perceived fluency. Importantly, as already observed in previous studies that have articulated phenomenological data with behavioral data (e.g., Gal-Petitfaux et al., 2013; R’Kiouak et al., 2016; Seifert et al., 2017), we were not necessarily expecting a fully congruent relationship between what had been meaningfully experienced and what had been behaviorally performed.

### Ethics Statement

The protocol followed the guidelines of the Declaration of Helsinki. Procedures were explained to the participant who then gave his written consent to participate.

## Results

The results are presented in three parts. The first part presents the characterization of the climber’s enacted fluency based on the thematic analysis of his course of experience. The aim of the thematic analysis was to characterize the climber’s definition of his enacted fluency. The second part presents the phenomenological dynamics of the climber’s enacted fluency (i.e., the tracking of the temporal emergence of the general dimensions) in association with his fluency scores in the unchanged environment. The third part presents the phenomenological dynamics of the climber’s enacted fluency in association to his fluency scores in the novel environments. The fluency scores values are presented in the Supplementary  Material.

### The Climber’s Enacted Fluency

#### Intentions

The results of the thematic analysis of the climber’s intentions revealed three general dimensions (Figure 1): (i) Seeking and maintaining balance while climbing, (ii) Ensuring the correct execution of movement chaining, and (iii) Improving the timing of the climb. The general dimension Seeking and maintaining balance while climbing referred to the climber’s concerns about avoiding losses of balance, which would have threatened climbing fluency. This intention involved either the whole body or the feet. The general dimension Ensuring the correct execution of movement chaining referred to the climber’s concerns about finding and correctly performing a chaining of movements and avoiding errors to ensure fluency. This intention involved either hand chaining or hand-foot chaining. The general dimension Improving the timing of the climb referred to the climber’s concerns about avoiding stops and saccades and pacing his movements with dynamic movements in order to improve fluency. This intention involved either hand movements or hand-foot synchronization.

#### Actions

The results of the thematic analysis of the climber’s actions revealed four general dimensions (Figure 2): (i) Carrying out the planned chaining, (ii) Making errors while climbing, (iii) Deliberately modifying the planned movements while climbing, and (iv) Adjusting the foot chaining while climbing. These general dimensions encompassed the climber’s meaningful actions during his climbs; it is important to note that for a given climb, several actions may have been performed simultaneously (e.g., carrying out the planned hand chaining while adjusting the foot chaining). The general dimension Carrying out the planned chaining referred to the climber’s actions corresponding to the movements he had planned during the preview. These actions concerned either the hand chaining or the hand-foot chaining. The general dimension Making errors when climbing referred to the climber’s unplanned actions, which he lived as errors. These meaningful errors involved either the hands (i.e., taking a wrong handhold or crossing the hands) or the feet (i.e., taking a wrong foothold or a foot slipping). The general dimension Deliberately modifying the planned movements while climbing referred to the climber’s modifications as he attempted to improve his climbing fluency. These modifications involved either hand chaining or hand-foot chaining. The general dimension Adjusting foot chaining while climbing referred to the climber’s actions linked to the hold-by-hold improvisation of the feet, meaning that he had not planned a detailed chaining for his feet during the preview as he expected that the feet would follow the hands. As a consequence, he systematically looked at his foot placement when moving from one hold to another, leading to improvisations, hesitations, or careful observations of the holds before using them.

#### Perceptions

The results of the thematic analysis of the climber’s perceptions revealed four general dimensions (Figure 3): (i) Sensation of being balanced, (ii) Sensation of being unbalanced balance, (iii) Sensation of efficient climbing timing, and (iv) Sensation of perturbed climbing timing. These general dimensions encompassed the climber’s meaningful perceptions during the climbs; it is important to note that for a given climb, several perceptions may have emerged simultaneously (e.g., efficient climbing timing and balance). The general dimension Sensation of being balanced referred to the climber’s positive perceptions of balance, such as the sensation that the plexus was aligned along the vertical axis or stability via the feet. The general dimension Sensation of being unbalanced referred to the climber’s negative perceptions of losing balance, such as uncomfortable, unstable, and unsafe positions of the body or feet, which perturbed the perceived fluency. The general dimension Sensation of efficient climbing timing referred to the climber’s positive and efficient perceptions of the climbing timing and contributed to the perception of good climbing fluency. This efficient timing was perceived at the level of the whole body displacement (i.e., no stops or saccades, sensations of being in constant movement, sensation of speed), the hand chaining (i.e., sensations of regular pace and dynamic tempo), or the hand-foot chaining (i.e., sensation of climbing a ladder, efficient synchronization, and tempo). The general dimension Sensation of perturbed climbing timing referred to the climber’s negative perceptions of the timing, which altered the perceived climbing fluency. This perturbed timing was perceived either through perturbations in the climbing fluency (i.e., stops, saccades, immobility, and perturbation in the chaining) or the sensation of inefficient slowness (i.e., sensations of climbing slowly and spending too much time on the holds).

The identification of these general dimensions, which made up the climber’s own world in his definition of enacted fluency, indicated that his definition of fluency was mainly linked to the notion of timing. Indeed, for intentions, 44 occurrences referred to improvements in timing, and for perceptions, 77 occurrences referred to the sensation of efficient or disturbed timing. This salience of timing was mainly actualized through the execution of a planned chaining (64 occurrences). In other words, according to the climber, climbing fluently means climbing with efficient timing - that is, no saccades, no stops, and being in constant movement.

### Dynamics of These Experiences in the Unchanged Environment

As we detail below, the analysis of the dynamics of the climber’s definition of his enacted fluency revealed the importance of climbing with efficient timing. We therefore focused on episodes (i.e., one trial or a succession of trials) where perturbations in the timing emerged in the climber’s perceptions (Figure 4C). We successively present: (i) the dynamics of the climber’s lived experience in the unchanged environment in association with the evolution of the four indicators of fluency (i.e., CT, percentage of immobility, GIE, and jerk) and (ii) the crucial episodes in which the timing of the climb was lived as perturbed.

#### Dynamics of the Climber’s Lived Experience on the CR

As shown in Figure 4A, in the unchanged environment (i.e., the CR), the general depiction of the dynamics of the intentions showed that the climber was mainly concerned with ensuring the correct execution of chaining (trials 1–21). It should be noted that trials 1–6 were characterized by the second-order theme Correctly performing the planned hand chaining before moving to the second-order theme, Correctly performing the planned hand-foot chaining (trials 7–21). Then a switch occurred in trial 22, where the intention to improve the climbing timing for the hand chaining emerged as salient, as characterized by the second-order theme Pacing the climb with dynamic hand chaining. Regarding his actions, while he mainly carried out his planned chaining (Figure 4B), he made errors at the beginning (trials 1, 2, and 4) and in trials 10, 11, 13, 15, and 26, which were accompanied by deliberate modifications in trials 3 and 20. The general dynamics of the perceptions show an entanglement of the sensations linked to balance and timing, made up of frequent switches from “positive” elements (i.e., balance and efficient timing) to “negative” elements (i.e., losses of balance and perturbed timing) and inversely (Figure 4C). We can observe that the issues linked to losses of balance (trials 1, 2, 13, and 15) were associated were errors in chaining, which gave rise to intentions about maintaining balance. The modalities through which perturbed timing was perceived changed over time: from sensations of disruptions in the climbing fluency because of stops and saccades (trials 4 and 10–12) to sensations of inefficient slowness of the hand chaining and the time spent on the holds (trials 22–24 and 27, 28). Similarly, the modalities through which efficient timing was perceived moved from the absence of stops and saccades (trials 5–9) toward sensations of efficient hand-foot synchronization (trials 16–19) and rhythm in the chaining (trials 25, 26, 29, and 30). Finally, the dynamics of his evolving fluency scores showed a global non-linear progression from performances in the third tercile (trials 1–5) to the second tercile, and then the first tercile in trials 16–19 and trials 27–30 (Figure 4D).

#### The Crucial Episodes of Perturbed Timing

The dynamics of the general dimensions of the climber’s experience during the 30 trials on the CR revealed four crucial episodes. These episodes were qualified as crucial because they were characterized by disruptions in the climbing timing, which were characterized as follows:

##### Episode 1, trial 4: an unplanned hand-crossing that disrupted the timing

Prior to this episode, the first two trials were characterized by errors in hand chaining during which the climber felt losses of balance before making a modification in trial 3. In trial 4, the climber had hand-focus intentions in the sense that he sought to perform correct hand chaining from the bottom to the top in order to avoid errors and losses of balance. However, after a fluent first section, he made an error with his hands in the middle of the route (i.e., he crossed his hands) and felt immobile. He characterized this error as disrupting the promising fluency he had felt at the start and was disappointed because he could not actualize his entire plan for the hand chaining. The error made in this climb was crucial for the climber as he explicitly built the knowledge that hand crossing was a threat to fluency and had to be avoided in the following climbs, as illustrated by the following excerpt from verbalizations on trial 4:

Climber: [points to the moment in the video where he found himself with crossed arms on two holds and the end of the route] “And here, I had expected to do a hand repetition [two successive handholds with the same hand] but at the end I was doing just anything, kind of unthinkingly like a robot.
Researcher: And suddenly, there you cross your arms.
Climber: Yes. That’s the mechanical side, I crossed them and that was a huge mistake. That brought my whole body to a standstill.
Researcher: So there, how were you feeling about the fluency at that point?
Climber: I was feeling fluent all along the route but at that moment where I made the mistake with my hand, I thought oh great now it’s over. There I knew that it wasn’t good … Having your arms crossed doesn’t feel good, but more than anything, it’s not at all practical … it gets really jerky. At least it helped me to know how to do things better the next time, to make up for mistakes like that in the next trials and avoid crossing my hands.

This episode was confirmed by poor fluency scores, as all indicators were in the third tercile.

##### Episode 2, trials 10–12: the feet that slow down the ascension

This episode characterized the switch from hand-focused intentions to hand-foot focused intentions, meaning that the climber was no longer exclusively concerned about correct hand chaining but was trying to improve his fluency by integrating the feet as part of the whole chaining. This switch to hand-foot chaining had been successfully initiated in trials 7–9 (he felt like he was climbing on a ladder and the scores moved to the second tercile in trial 9). However, during this episode, while he attempted to keep the same hand-foot chaining, he made an error in the hand chaining in trial 10 and an error in the foot chaining in trial 11 despite his attempt to stick to his planned hand-foot chaining: “When I finished it, I thought that my fluency on that route was zero because there were moments where I stopped, small hesitations with my feet. In any case, I thought it was no good. So it showed me where I needed to be careful in the next trials” (trial 10). These errors led to sensations of lacking rhythm and being stuck on the holds, which negatively impacted his assessment of his climbing fluency: “I don’t feel like I was very successful on that route … I don’t know, there was no flow, I felt slow” (trial 11). This negative perception of fluency was confirmed by the scores (all are in the third tercile, except for the IR which was in tercile 2 in trial 10). In trial 12, he recited his planned hand-foot chaining but still had the sensations of being slow and spending too much time on the holds. In spite of these sensations, the scores for the GIE, jerk, and CT improved.

##### Episode 3, trials 22–24: the sensation of inefficient slowness

In this episode, the climber was no longer concerned about his hand-foot chaining but instead he sought to improve his climbing timing by pacing his climb with dynamic hand chaining; “I’m thinking that I have to be more dynamic so I don’t get stuck on the handholds, I have to move from one to another. I rehearse with my hands, I know the chaining by heart, I don’t want to bother with the feet because I have the impression that that had an impact, thinking about my feet, and so I rehearse with my hands so I don’t get upset … So I want to be hyper-dynamic, just with my hands” (trial 24). During the climbs, the climber recited his well-known hand chaining without errors or modifications but had the sensation of climbing slowly (trials 22 and 23) and spending too much time on the holds, and so he assessed his fluency as poor: “My whole body is slow, I can’t keep up a steady rhythm, there’s always a moment of slowdown, of kind of floating and so it’s not fluent … and every time it stops because I look down to make sure I don’t mess up with my feet. I wanted to be dynamic but I wasn’t.” Although he perceived his fluency as poor in this episode, these negative perceptions were contrasted by the scores, which moved to terciles 2 and 1, especially for trial 24 (all indicators reached tercile 1).

##### Episode 4, trials 27–28: saccades and compensation

In this episode, the climber knew his hand chaining by heart (he was no longer concerned with the feet chaining, he just expected them to “follow” the pace imposed by his hands), and sought to improve his climbing fluency by pacing his climb with dynamic hand chaining. However, during trial 27, one of his feet slipped from a hold and he had to compensate by pulling with his arms to restore his position and finish his climb. At this moment, the climber felt a saccade that perturbed his fluency but was relieved that he had managed to maintain fluency with his hands: “My foot slipped on the hold just when I pushed on it … I was so mad! I fixed it by pulling with my arms … the fluency wasn’t too bad, except for the foot that slipped and caused me to jerk.” In trial 28, he had the same intention of putting dynamism in the hand chaining and recited his planned chaining, but then felt that he was slow when grasping the holds, as he felt a small saccade before each handhold: “Fluency is not really that, there’s a little jerk of the hands when I grab the holds, my hands slow on each hold ….” This episode was characterized by good fluency scores in tercile 1, in contrast to the climber’s experience.

### Dynamics of the Climber’s Enacted Fluency in the Novel Environments

The analysis of the timing perturbations that were meaningful to the climber enabled us to identify four ways of experiencing novelty: novelty without perturbation (variants 2 and 3), novelty without immediate perturbation (variants 4 and 5), novelty as a source of punctual perturbation (variants 1 and 8), and novelty as a source of recurrent perturbation (variants 6, 7, and 9).

#### Novelty Without Perturbation

This way of experiencing novelty was identified in variants 2 and 3 (Figure 5). In the first trial on variant 2, the climber sought to maintain his balance and chain his hands correctly (Figure 5A, left). He carried out his chaining while simultaneously looking at the foot placement (Figure 5B, left). He had positive perceptions of ease and efficient timing (i.e., no stops, no saccades, and constant movement of the body) (Figure 5C, left) and was pleased. In the following climbs, he no longer focused on the correctness of his chaining but instead on balance and improving his timing by putting dynamism into his hand routine, as illustrated by the following excerpt: “I know the chaining now, so what’s important is to make it dynamic … so it has to flow, no more losing time” (Variant 2, trial 2). While performing his routine in trials 2–6, he also felt an efficient timing (i.e., no stops, no saccades, and constant movement of the body). His fluency scores showed an improvement in the temporal indicators (i.e., from tercile 2 to 1 for the IR, from tercile 3 to 2 for the CT and the jerk) while the spatial indicator (i.e., the GIE) remained in tercile 3 for all the climbs (Figure 5D, left).

In the first trial on variant 3, the climber’s intentions were also linked to climbing rapidly while maintaining balance and correctly chaining the hands (Figure 5, right). He carried out his hand plan and adjusted his foot placement at the same time (Figure 5B, right). He felt sensations of climbing fast with no stops in his climbing pace (Figure 5C, right). In trial 2, while he was focused on maintaining the correct execution of hand chaining, he modified the foot chaining and felt a better pace. He validated this change for the subsequent trials by integrating his feet into the planned chaining. He felt an efficient hand-foot synchronization that he expressed as “climbing like on a ladder.” His scores were mainly in terciles 1 and 2, and in tercile 3 for the GIE in trial 1 and immobility in trial 4. In sum, he discovered that he could use his feet to improve fluency by synchronizing their movements with the hand chaining (i.e., he felt a better climbing pace that he described as “climbing like on a ladder”) and showed a tendency to a convergence between his perceived fluency and the scores (Figure 5D, right).

The climber’s way of acting in these environments – where he experienced no perturbation of the timing of his climbs – consisted of elaborating a chaining plan and ensuring its correct execution to guarantee efficient timing. The attempts to carry out his plans were not always accompanied by high fluency scores.

#### Novelty With Delayed Perturbations

This way of experiencing novelty was identified in variants 4 and 5 (Figure 6). During the first three trials on variant 4, the climber sought to climb this route like a ladder by pacing his planned hand-foot chaining to improve his timing (Figure 6A, left). He recognized that the spatial layout of the holds would let him climb the route “like a ladder” and expressed his intentions to do so: “I’m thinking to do right arm, left arm, right arm, left arm so it seems like a ladder (he rhythmically mimes the left–right alternations), I’m looking for the rungs [of the ladder]. So finally, I try to climb like I’m on a ladder” (variant 4, trial 1). He carried out his plan and felt an efficient hand-foot timing (Figures 6B,C, left). He started trial 4 with the intention of maintaining his balance, ensuring the correct execution of his planned hand-foot chaining, and improving the timing through synchronized hand-foot movements. He nevertheless made an error in the foot chaining and slipped, causing a perturbation to his perceived hand-foot timing. The same scenario occurred in trial 5, where he made an error in the hand chaining, which unbalanced him and also perturbed his hand-foot timing. After trials 4 and 5, he concluded that integrating the feet in the elaboration of a chaining was not efficient for fluency because it increased the possibility of avoidable errors and stops. Therefore, in trial 6, he decided to focus exclusively on his hand chaining, which he carried out while adjusting the foot chaining at the same time, as he had no prior plans for them. By doing so, he stopped less often and again felt efficient timing. All his scores were in tercile 1, except for trial 5, in which the IR was in tercile 3 and the CT, jerk, and GIE were in tercile 2 (Figure 6D, left).

In the first five trials on variant 5, he sought the sensations of climbing a ladder by purposely and exclusively focusing on the timing of his hands (Figure 6A, right). He carried out the planned hand chaining and adjusted his feet (Figure 6B, right). He felt a regular rhythm in his movements as well as an efficient hand-foot alternation and balance (Figure 6C, right). In trial 6, he also carried out the hand chaining while adjusting the feet at the same time but he felt saccades that negatively impacted his perception of fluency. His scores were mainly in terciles 1 and 2 and the GIE of trial 4 and the IR of trial 5 were in tercile 3 (Figure 6D, right).

In sum, for each first trial on variants 4 and 5, the climber had direct intentions to improve the timing of his climbs. The ladder metaphor was useful for anticipating the hand-foot chaining and timing in the aim of improving climbing fluency, but it also led to errors and balance losses that perturbed the timing. As a consequence, the climber decided to focus exclusively on his hands, and he carried out the planned-on chaining while adjusting the foot chaining at the same time, as there was no prior plan for them. Overall, the way of metaphorically living his climb as he looked for efficient timing was confirmed by high fluency scores, and the perceived errors and perturbations were confirmed by poorer scores.

#### Novelty as a Source of Punctual Perturbation

This way of experiencing novelty was identified on variants 1 and 8 (Figure 7). In the first trial on variant 1, the climber sought to maintain his balance and correctly chain his hands (Figure 7A, left). He deliberately modified this chaining while climbing and had the sensation that his whole body was in constant movement (Figures 7B,C, left). In trial 2, he carried out the hand chaining, including the modification he had made in the previous climb, and perceived his timing as efficient. In trial 3, he sought to climb faster to improve his fluency. He executed his hand chaining and adjusted his feet at the same time. He again had the sensation of efficient timing (i.e., no stops, saccades, or hesitations). In trial 4, he sought to reproduce the same climb but he modified his hand chaining and felt stops that perturbed his timing. In trial 5, he carried out his initial hand chaining and felt speed and balance. In trial 6, he was no longer concerned with sticking to his plan for chaining but rather sought sensations of balance and speed. He executed his plan and had good sensations of timing and balance. In sum, while he progressively constructed his chaining with modifications throughout the trials, he felt a progressive improvement in his fluency (i.e., body in constant movement, speed, and balance). Despite his promising sensations of timing, his scores were all in tercile 3 for each trial (Figure 7D, left).

In the first trial on variant 8, he made errors in hand chaining (Figure 7B, right). He felt that his chaining was completely perturbed and had the sensation of being stuck on the route, which perturbed his timing (Figure 7C, right).

Researcher [at the beginning of the climb]: So there you take off, you do left hand, right hand.
Climber: There, I put out my right hand, the worst thing to do. So suddenly I didn’t know what to do to untangle myself so I started thinking … I said, oh boy this is going to be complicated, what am I going to do … Suddenly, I sent my right hand up somehow … but right away, it missed and it was a mess and I had no backup plan in case it didn’t work … so fluency level: zero … everything I had planned to do was completely perturbed, a complete failure because then I started to hesitate and I just got blocked. So for the next climb, I thought just put your left hand there at the beginning and after that, it’ll be easy. The key is the start (variant 8, trial 1).

This perturbation was confirmed by the scores, which were all in tercile 3 in the first trial (Figure 7D, right). In the following five trials, he sought to avoid the error in chaining and put dynamism in his hand chaining. He recited his planned chaining and felt he was dynamic, with an efficient timing and fluency. Congruently, his scores were in terciles 1 and 2 in the other trials (Figure 7D, right).

In sum, in these two variants, the climber constructed his chaining through punctual errors and modifications, which helped to build, improve, and optimize his chaining until he could look for efficient timing. This search for timing efficiency was either never confirmed by high scores (cf. variant 1) or followed a tendency to progression (cf. variant 8).

#### Novelty as a Source of Recurrent Perturbation

This way of experiencing novelty was identified on variants 6, 7, and 9 (Figure 8). On variant 6, the climber reported that the global impression that his climb was not well integrated (i.e., sensations of spending too much time on the holds and inefficient slowness). In trial 1, he modified his planned hand chaining while climbing and had the sensations of spending too much time on the holds and inefficient slowness (Figures 8B,C, upper left). While he carried out his plan in trial 2, he still had the same sensations that perturbed his timing. In trial 3, he made an error in the hand chaining that made him feel that he was moving slowly and taking too long. In trial 4, he modified his chaining and felt better sensations of timing (i.e., no stops and constant rhythm). In trials 5 and 6, he recited the modified plan and again had positive sensations of efficient timing. While he perceived progressive improvements in his fluency from trials 1 to 3 and 4 to 6, his scores did not reflect this as most of his temporal scores were in tercile 3 (except for the IR in trial 3, which was in tercile 1) and the spatial indicator (GIE) was between terciles 1 and 2 (Figure 8D, upper left).

On variant 7, although he recited his planned hand chaining in all the trials (Figure 8B, upper right), he had several hesitations with his foot placement from trials 1 to 4 that perturbed his timing in the sense that he felt his feet were “braking” the hand pace (i.e., he had the sensation of spending a long time finding footholds, slowness, and even some stops):

Climber: I was hesitating a lot with my feet, I had to really look for the footholds. Suddenly I was blocked with my hands.
Researcher: So if I understand you, chaining the hands was fine but the feet were a problem. When this happens, how do you feel the fluency?
Climber: Well, I have the sensation that time has slowed down. I’m spending time on the holds, so I don’t feel fluent at all. My feet really slowed me down.

Hence, he intended to improve his timing with dynamic hand chaining (Figure 8A, upper right). In trials 5 and 6, he also recited the chaining while also adjusting his feet and felt more dynamic. His scores progressively improved from tercile 3 to tercile 1 throughout the trials (Figure 8D, upper right).

In the first trial on variant 9, the climber created a new chaining, which he then modified while climbing (Figure 8B, lower left). While making this modification, he felt stops and small hesitations (Figure 8C, lower left). In trial 2, he focused on reproducing the hand chaining. He recited it while looking at his feet and he also felt his feet were slow. In trial 3, he sought to climb dynamically but he made an error with his hands, his feet slipped, and he felt bad sensations about the timing. In trials 4 and 5, he carried out the planned hand chaining and had better sensations of pace. In trial 6, he sought speed in his hand chaining; while he recited the chaining, he felt slow and jerky. His scores were mainly in tercile 2, except for the CT in trial 1 and the GIE in trials 1 and 2, which were in tercile 3 (Figure 8D, lower left).

In sum, on these three variants, the climber constructed his chaining through errors and modifications but he never managed to feel efficient timing. While the fluency scores were overall poor, in one case the scores matched the sensation of inefficient timing (cf. variant 6), and in another case (variants 7 and 9), the improvement in the fluency scores was not perceived as such.

## Discussion

The aim of this case study was to analyze the dynamics of a novice climber’s lived experience and performances during a 10-session training protocol of different climbing tasks. He was confronted with both unchanged and novel routes and was instructed to climb as fluently as possible. Our analysis methodology focused on integrating the climber’s experience with his “objectivized” progression, which enabled us to obtain a detailed depiction of what was meaningful for him during the learning protocol. In other words, we sought to characterize the climber–environment coupling from what was meaningful for him. This methodology enabled us to highlight what we termed a phenomenological synthesis, which refers to the way an actor deals with the complexity of the interaction between his activity and the learning environment.

Our results are summarized around the following points: (i) the climber’s construction of significations in terms of his intentions, actions, and perceptions that defined how he enacted his fluency in timing; (ii) the non-linear progression of his performance in the unchanged environment and the crucial episodes in his experience; (iii) the four ways of experiencing the novel environments that were identified: novelty without perturbation, novelty with delayed perturbation, novelty as a source of punctual perturbation, and novelty as a source of recurrent perturbation); and (iv) the climber’s perception of fluency, which was not systemically matched by his performance scores. Our discussion focuses on the following points: (i) the enacted fluency as a phenomenological synthesis of the climbing activity, (ii) the emergence and definition of the phenomenological synthesis, and (iii) the dynamic property of the phenomenological synthesis.

### The Enacted Fluency as a Phenomenological Synthesis of the Climbing Activity

The enactive paradigm conceives the relation between an actor and the environment as a structural and dynamic coupling (Varela et al., 1991). This means that the climber considered the complexity of the significations he was building through his intentions, actions, and perceptions. He defined, from his point of view, the learning environment: the material and spatial layout of the climbing routes, the task instructions (i.e., to climb as fluently as possible), and the fluency scores. We suggest that the climber summarized this complexity through a phenomenological synthesis. This synthesis expressed his specification of his own world and it is probable that another climber would have had another phenomenological synthesis.

To face the task requirements in the two types of environments (unchanged and novel routes), the climber created his own definition of fluency, which was expressed through the presence/absence of balance, stops, saccades, execution of the planned hand chaining or hand-foot chaining, and climbing timing. His definition of fluency was specifically highlighted by the results of our thematic analysis (Figures 1–3), which showed that he expressed his concern about improving the timing of his climbs by carrying out the movements that he assessed as efficient. The enacted fluency was embedded in the climber’s experience as intentionality (i.e., expectations, concerns, and goals), embodiment (actualization through his meaningful actions, multi-modal perceptions), and sense-making (i.e., the construction of meaning and the knowledge built from the interactions with the learning situations). On the basis of these results, we discuss the emergence and definition of this phenomenological synthesis and its dynamic property. Indeed, as we will detail below, this phenomenological synthesis enabled the climber to assess, adjust, explore, and create chaining, which participated in re-defining this synthesis.

### Emergence and Definition of the Phenomenological Synthesis

Our results show that the emergence and definition of the phenomenological synthesis during the first interaction with a learning environment is stimulated either by links to past experiences or by the actor’s perceptions and appropriation of the learning environment. In our study, the climber referred to past experiences that he assessed as similar in this environment, giving rise to what we qualify as the effect of metaphor (i.e., climbing a ladder). This effect characterized his coupling with the environment and the use of pre-existing coordination (i.e., the movement of climbing a ladder), which refers to a pre-built chaining. For example, on variants 4 and 5 (Figure 6), the climber directly engaged with these novel environments with the intention to efficiently synchronize his hands and feet because the spatial layout of the holds suggested that he perform his chaining as if he were on a ladder. This metaphorical effect might lead to a satisfaction or new perturbations, which would participate in re-defining the phenomenological synthesis. Although the metaphorical effect enabled the climber to engage directly with expectations linked to efficient fluency, it did not preclude the emergence of errors. Indeed, in trial 4 on variant 4, he made an error in foot chaining, which had repercussions for the hand chaining and consequently he assessed his fluency negatively, which was confirmed by the scores.

In other cases, the recognition of similarities with past experiences did not emerge from the coupling with the environment. Rather, it was the significations built by the climber at that moment that participated in defining his phenomenological synthesis to act in a novel environment. For example, the first few times the climber climbed the CR (i.e., a new route), he made errors in trials 1 and 2, made a modification in trial 3, and made another error in trial 4, before carrying out his planned chaining from trials 5 to 9 (Figure 4). However, as observed on both CR and the variants, the execution of his plans did not prevent him from making errors or modifications, which generated further modifications and transformations of the phenomenological synthesis. Our results for all the variants showed that meaningful perturbations emerged at different moments in the task (i.e., delayed and no perturbation) in different forms (i.e., punctual or recurrent perturbation), highlighting the unpredictable, non-prescriptive, and autonomous properties of activity. In what follows, we will consider the extent to which the phenomenological synthesis was under perpetual modification, thus dynamically rebuilding the climber’s own world.

### The Dynamic Property of the Phenomenological Synthesis

Our results reveal the non-linearity of the climber’s lived experiences on both the CR and the variants. This non-linearity was linked to the dynamic property of the phenomenological synthesis, as the performance of a memorized chaining did not preclude the emergence of errors (i.e., described as such by the climber) that perturbed this synthesis, causing him to lose his balance, stop his progress, and display jerky behavior, thereby perturbing his timing. These perturbations led to adjustments in the phenomenological synthesis for the subsequent trials. For example, the error he made during episode 1 on the CR (i.e., he crossed his hands and was blocked in his ascension) prompted him to avoid crossing his hands in the following climbs and hence modify his movement sequence. In trial 5 on variant 4, he made an error in his hand chaining because of inefficient foot placement; this error had direct repercussions on the following trial on variant 4 and in all trials on variant 5, as he decided to abandon the hand-foot synchronization to focus exclusively on his hand chaining (Figure 6). Thus, meaningful errors may explain adaptations in learners’ intentions and actions, as, for example, in this case: errors prompted the climber to return to previously mastered movements and temporally stop exploring other behaviors.

The dynamic property was identified in our study through two effects: challenge and the refinement in perceptions. Being repeatedly successful in the task (i.e., absence of meaningful perturbation in the enacted fluency) might have prompted the climber to undertake even more challenging actions, which would then encourage the dynamic transformation of the phenomenological synthesis. We have termed this phenomenon the challenge effect. The climber consistently sought to perform the task better, and it is fair to assume that being informed of his scores at the beginning of each session motivated him to try to improve them. After having successfully carried out the same chaining several times, he challenged himself by integrating his feet into the chaining to improve fluency. As shown in Figure 4, by adding more body segments to his plans for chaining, he made the task more complex, with the challenge effect characterized by these attempts to synchronize the hands and feet to improve his fluency (see episode 2, trials 10–12). The results show the challenge effect especially on the CR, because the sheer number of climbing repetitions enabled him to (i) turn the execution of successful chaining into a routine and (ii) regularly consult his prior scores, and thus build knowledge about his performance on this route. Relatedly, through the challenge effect, the learner may well have incorporated prompts and task constraints during the repetitions and was thus encouraged to explore new behaviors to meet the expectations even better. However, the challenge effect did not systematically lead to viable modifications in the chaining. Indeed, after the perturbations induced by the challenge, the climber returned to hand chaining, which had previously worked for him. The challenge effect can be linked to the results of studies highlighting intermittent regimes in learning dynamics (Nourrit et al., 2003; Teulier and Delignières, 2007). It can also be assumed that the challenge effect does not emerge for every learner: for example, Orth et al. (2018) observed no progress for some of their participants in a climbing task, as these participants made sure their climbs would be successful by repeating a well-practiced chaining rather than exploring new solutions. The lack of exploration while learning was also reported by Chow et al. (2008): a participant displayed little movement variability and a slower rate of progress. From this perspective, the presence/absence of the challenge effect in the phenomenological data might provide clues to understanding the emergence of new behaviors or the stagnation that comes with repeating the same behaviors.

Throughout the CR trials, the climber’s intentions, actions, and perceptions (which constitute the phenomenological synthesis) were transformed in the sense that the timing perturbations were lived differently between the first trials and the last trials, highlighting what we would define as an effect of a refinement in perceptions. Indeed, while the climber did not refer to the time spent grasping each handhold in the first trials on the CR, he perceived and verbalized about these times in the last trials when they seemed to last too long or were hesitant or jerky: he perceived them as sources of perturbation in his fluency. The refinement was observable in crucial episodes: in episode 1 (trial 4), the climber synthesized his perception of fluency through stops and losses of balance that involved his whole body, and in episode 4 (trials 27–28), he synthesized his perception of fluency through sensations of small saccades when the hands were approaching the holds (Figure 4). In episode 1 on the CR, the climber perceived the saccades through stops of the whole body and in episode 4 on the CR he perceived the saccades through body parts, mainly the hands before grasping each handhold. On this occasion, the “hesitation” he felt in the hands was lived as a perturbation to fluency. The idea of refining the phenomenological synthesis is congruent with the results of studies of expertise in sports, which have noted that experts have finer sensations than beginners when interacting with artifacts (Gal-Petitfaux et al., 2013; Adé et al., 2017).

From a phenomenological point of view, the dynamics of the climber’s live experience revealed that the effect of the refinement in his perceptions was not necessarily reflected in the scores. Indeed, while the dynamics of the lived experience provided insight into some of the perturbations identified at the behavioral level, they also revealed meaningful perturbations that were not reflected in the scores. In this sense, the progressive refinement in perceptions seemed to lead to divergences with the objectivized fluency assessed with stable behavioral indicators (i.e., the CT, the GIE, the IR, and the jerk). As illustrated in Figure 4, in episode 1, the poor scores were convergent with the synthesis of the fluency (i.e., stops and loss of balance). In episode 2, this relationship was also convergent, but the scores indicated a tendency toward improvement. In episodes 3 and 4, the scores contrasted with the climber’s poor perception of fluency, revealing a divergent relationship between the climber’s synthesis and the behavioral assessment. These observations suggest that this progressive divergence between lived experience and performances might have been linked to a certain level of task expertise, in the sense that the climber – even if he lived punctual perturbations – achieved a certain level of fluency as he no longer performed inefficient actions that negatively impacted the scores. This divergence between the dynamics of the phenomenological and behavioral data has been highlighted in other studies (e.g., Gal-Petitfaux et al., 2013; R’Kiouak et al., 2016; Hauw et al., 2017; Seifert et al., 2017) and it provides meaning to the behavioral output in a relation of mutual enrichment (Sève et al., 2013; Depraz et al., 2017; Rochat et al., 2019).

Taken together our findings argue for a phenomenological synthesis between the actor and the environment, in line with theoretical hypotheses about activity in learning (Seifert et al., 2016; Adé et al., 2018; Petiot and Saury, 2019). This synthesis is thus a part of the actor’s activity, which constantly participates in re-defining his own world. In this sense and as shown by our results, this phenomenological synthesis is inseparably operatory and dynamic.

## Conclusion and Further Perspectives

Our findings enabled us to conceive and validate a methodology that characterized the dynamics of a learner’s lived experiences in relation to the dynamics of his behavior in a learning environment of variable practice. Our results also confirmed and enriched the literature on the enactive conception of learning in physical education and sport (Masciotra et al., 2008; Saury et al., 2013; Hauw, 2018). Indeed, in line with non-linear pedagogies (Chow, 2013), which give much importance to individual pathways in dealing with environmental constraints, the enactive conceptions put the learner’s lived experiences as they relate to environmental characteristics at the center of interest. Hence, they have the potential to provide solutions to the issues encountered in studies that use behavioral approaches exclusively. For example, they might provide insight into why some study participants following a given learning protocol do not exhibit the same tendency toward progression as others. Recent studies have underlined that it is important to design learning interventions that help practitioners to take into account learners’ lived experiences in learning situations (Durand, 2011; Saury et al., 2013; Adé and Komar, 2018). In this perspective, our study contributes to stimulate the timely reflections that consider the learners’ lived experiences as central. As a proposal for further research in sport pedagogy and teaching and in view of our results, we encourage researchers and practitioners to work and reflect on two following ideas. First, to consider learning as a reciprocal transformation: when an actor learns, he/she engages in a process in which he/she transforms himself/herself as well as his/her relationship to the learning environment. Second, to consider learning as a semiosis: each learner – through his/her interaction with the learning environment – brings forth his/her own world and lives his/her own meaningful situation. Hence, learning is enacting significations.

## Data Availability Statement

The datasets generated for this study are available on request to the corresponding author.

## Ethics Statement

Ethical review and approval was not required for the study on human participants in accordance with the local legislation and institutional requirements. The participant provided written informed consent to participate in this study.

## Author Contributions

LS designed the study. GH conducted the experiments, and collected and processed the behavioral data. NR and CG collected the phenomenological data. NR, CG, and DA processed the phenomenological data. NR and DA co-wrote the manuscript drafts. CG, GH, DH, PI, and LS reviewed the versions of the manuscript and provided critical feedback.

## Conflict of Interest

The authors declare that the research was conducted in the absence of any commercial or financial relationships that could be construed as a potential conflict of interest.

## Footnotes

1 http://www.luxov-connect.com/en/products/#touch
