# Supplementary material for: Dynamics of Experience in a Learning Protocol: A Case Study in Climbing
Source: Front Psychol. 2020 Feb 20;11:249. doi: 10.3389/fpsyg.2020.00249 (PMC7044343; doi:10.3389/fpsyg.2020.00249)
Supplement: Supplementary file 1 [file Data_Sheet_1.PDF]

Supplementary Material

Table 1. Means and standard deviation of the scores on the control route and the variants.

Control route				
	**GIE**	**Jerk (LN)**	**Immobility Ratio (%)**	**Climbing Time (s)**
**Mean**	0.80	12.12	20.90	14.16
**SD**	0.18	1.28	8.96	6.17
**Variants**				
**Mean**	0.75	11.96	18.48	12.89
**SD**	0.14	0.93	6.87	3.77

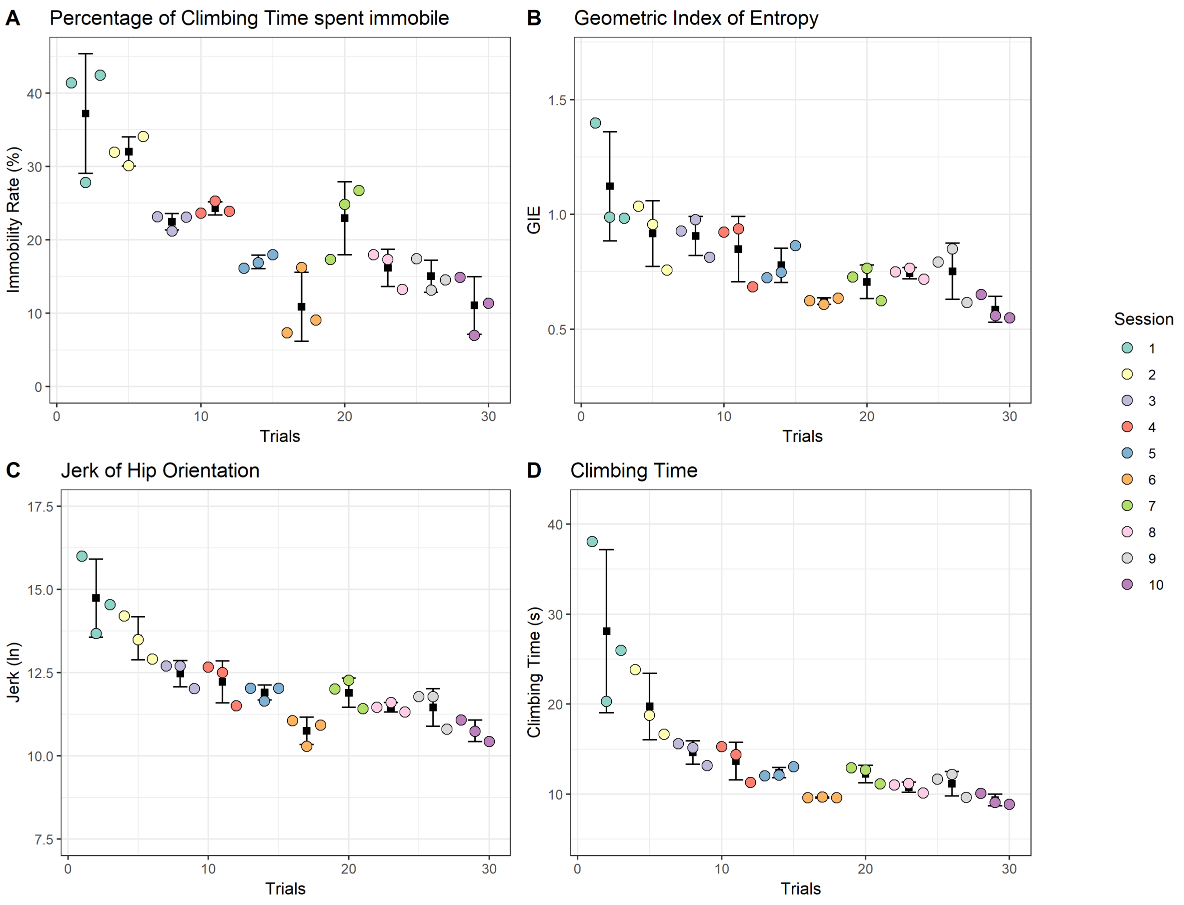

Figure 1. Fluency scores on the control route. The colors refer to the sessions. The black squares represent the mean of the three trials performed during the same session and the associated error bars represent the standard deviation

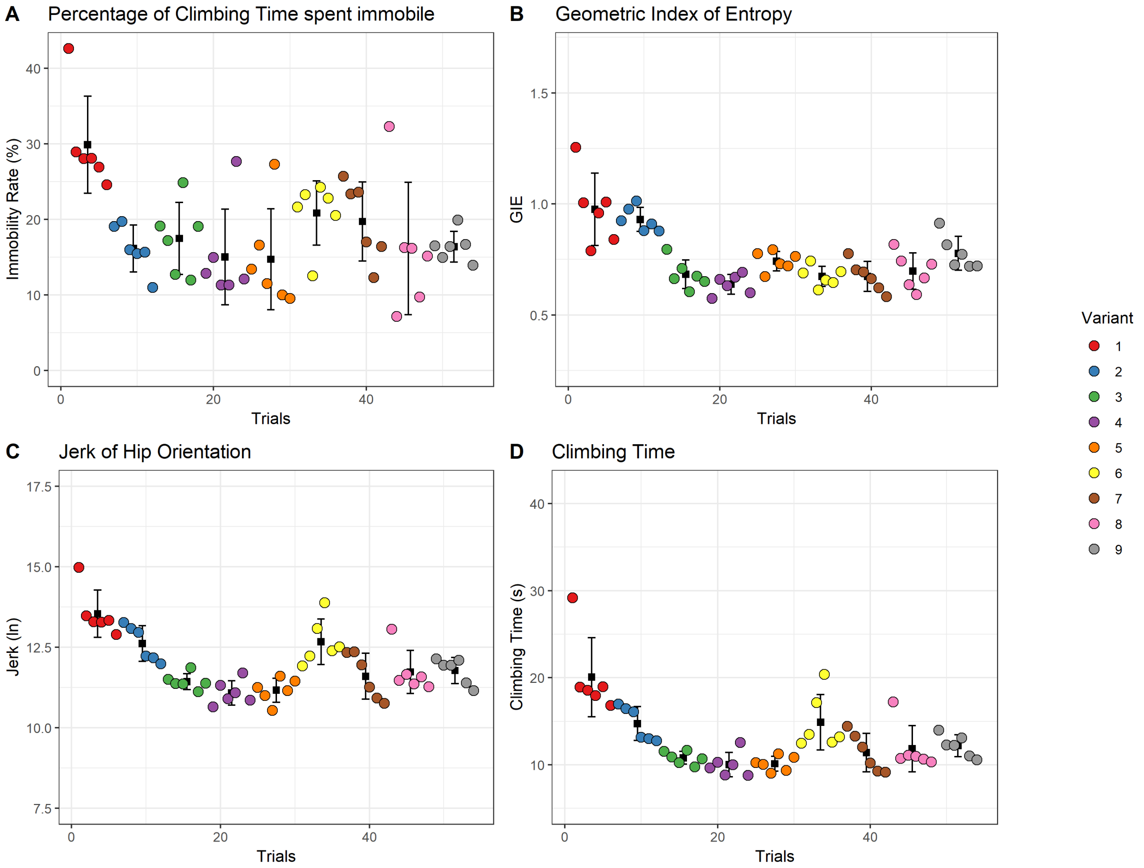

Figure 2. Fluency scores on the variants. The colors refer to the variants. The black squares represent the mean of the three trials performed during the same session and the associated error bars represent the standard deviation
